# Calpain inhibition mediates autophagy-dependent protection against polyglutamine toxicity

**DOI:** 10.1038/cdd.2014.151

**Published:** 2014-09-26

**Authors:** F M Menzies, M Garcia-Arencibia, S Imarisio, N C O'Sullivan, T Ricketts, B A Kent, M V Rao, W Lam, Z W Green-Thompson, R A Nixon, L M Saksida, T J Bussey, C J O'Kane, D C Rubinsztein

**Affiliations:** 1 Department of Medical Genetics, Cambridge Institute for Medical Research, University of Cambridge School of Clinical Medicine, Wellcome Trust/MRC Building, Cambridge Biomedical Campus, Hills Road, Cambridge CB2 0XY, UK; 2 Department of Genetics, University of Cambridge, Downing Street, Cambridge CB2 3EH, UK; 3 Department of Psychology, University of Cambridge, Cambridge, UK; 4 Translational and Cognitive Neuroscience Laboratory, MRC and Wellcome Trust Behavioural and Clinical Neuroscience Institute, University of Cambridge, Cambridge, UK; 5 Center for Dementia Research, Nathan S. Kline Institute, Orangeburg, NY, USA; 6 Department of Psychiatry, New York University Langone Medical Center, New York, NY, USA; 7 Department of Cell Biology, New York University Langone Medical Center, New York, NY, USA

## Abstract

Over recent years, accumulated evidence suggests that autophagy induction is protective in animal models of a number of neurodegenerative diseases. Intense research in the field has elucidated different pathways through which autophagy can be upregulated and it is important to establish how modulation of these pathways impacts upon disease progression *in vivo* and therefore which, if any, may have further therapeutic relevance. In addition, it is important to understand how alterations in these target pathways may affect normal physiology when constitutively modulated over a long time period, as would be required for treatment of neurodegenerative diseases. Here we evaluate the potential protective effect of downregulation of calpains. We demonstrate, in *Drosophila*, that calpain knockdown protects against the aggregation and toxicity of proteins, like mutant huntingtin, in an autophagy-dependent fashion. Furthermore, we demonstrate that, overexpression of the calpain inhibitor, calpastatin, increases autophagosome levels and is protective in a mouse model of Huntington's disease, improving motor signs and delaying the onset of tremors. Importantly, long-term inhibition of calpains did not result in any overt deleterious phenotypes in mice. Thus, calpain inhibition, or activation of autophagy pathways downstream of calpains, may be suitable therapeutic targets for diseases like Huntington's disease.

Huntington's disease (HD) is a currently incurable, autosomal dominant neurodegenerative disease resulting from the expansion of the trinucleotide (CAG) repeat region of the huntingtin (HTT) IT15 gene, encoding huntingtin protein (Htt). In mutant Htt, the polyglutamine tract encoded by this region contains over 35 glutamines and the length of the tract correlates inversely with the age of disease onset, with longer tracts resulting in earlier onset (reviewed in Imarisio *et al.*^1^). HD is one of the 10 trinucleotide repeat disorders resulting from expansions of polyglutamine tracts in different proteins. These expansions cause disease by conferring toxic gain-of-function properties onto the mutant proteins. Hence, one strategy that has been considered for HD and related diseases is to find ways of decreasing the levels of the mutant protein, for instance by harnessing the cell's capacity to degrade such aggregate-prone proteins via (macro)autophagy.^2, 3, 4, 5^ Autophagy involves the engulfment of cytoplasmic contents by double-membraned autophagosomes, which then traffic to lysosomes where their contents are degraded. Mutant huntingtin, some other polyglutamine expanded proteins like mutant ataxin 3, and proteins like tau (which mediates toxicity in Alzheimer's disease and related dementias) are autophagy substrates and their clearance can be enhanced in *Drosophila* and mouse models by autophagy upregulation, which also reduces their toxicity.^2, 3, 4,6^

Calpains are a family of calcium-activated cysteine proteases (reviewed in Ono and Sorimachi^7^) that inhibit autophagy. Strategies that reduce calpain activity in cell culture increase autophagy and decrease levels of autophagy substrates, like mutant Htt. These effects are likely to be mediated by Gsα, a heterotrimeric G-protein subunit which is activated by calpain cleavage. Similar to calpain inhibition, siRNA knockdown of Gsα, or chemical inhibition by NF449, induces autophagy and decreases the number of aggregates resulting from the overexpression of exon-1 Htt with an expanded polyglutamine repeat region (HttQ74) in cell culture models.^8^ In addition to this mechanism of autophagy upregulation by calpains, the core autophagy protein ATG5 has also been demonstrated to be cleaved and inactivated by calpains,^9,10^ suggesting that calpains may act on a number of substrates to negatively regulate autophagy.

In mammals, the two most abundantly expressed calpains are *μ*-calpain and m-calpain, which differ in their affinity for calcium and therefore the calcium concentration required for their activation. As well as being regulated by calcium, they are also controlled by an endogenous inhibitor, calpastatin (CAST). *Drosophila* have four forms of calpain:^11^ C*alpA* and *CalpB* are the conventional calpains formed by a recent duplication in the *Drosophila* insect lineage, *CalpC* is also an evolutionarily recent, but not highly conserved duplication (data not shown) and is thought to be catalytically inactive,^11^ and *CalpD* (SOL) is a member of the unconventional family of calpains. *Drosophila* does not appear to have any obvious orthologs of CAST.

A role for calpains in HD has been investigated previously. Following observations that shorter Htt fragments are more toxic than full-length Htt,^12^ it was demonstrated that Htt can be cleaved by both caspases^13^ and calpains^14^ to generate these toxic, short fragments. Blocking Htt cleavage by calpains by mutating their calpain cleavage sites decreases Htt aggregation and toxicity.^15^ In addition, calpain activation has been shown to be increased in HD patients compared with controls.^14^

In this study, we have investigated a role for calpain activity as a modulator of autophagy in both *Drosophila* and mouse models of HD. To avoid confounding effects from alterations in cleavage of Htt by calpain, we have used models expressing short fragments of Htt, which do not contain calpain cleavage sites and correspond to the shortest fragments of huntingtin seen in patients.^16^ We demonstrate that knockdown of *CalpA* in *Drosophila* is sufficient to both reduce the number of Htt aggregates and the toxicity associated with the expression of the mutant protein. Importantly, we show that these effects are autophagy-dependent. Furthermore, we show that overexpression of CAST in mice results in enhanced autophagy and improves locomotor function and delays tremor onset in a mouse model of HD, as well as decreasing the number of Htt aggregates seen in the brain. We extended the analysis of CAST overexpressing mice to investigate the possible adverse effects from long-term calpain inhibition or autophagy upregulation but did not observe any obvious deleterious effects.

## Results

### Calpain knockdown is protective in *Drosophila* expressing mutant huntingtin and protection requires functional autophagy

**Figure 1:**
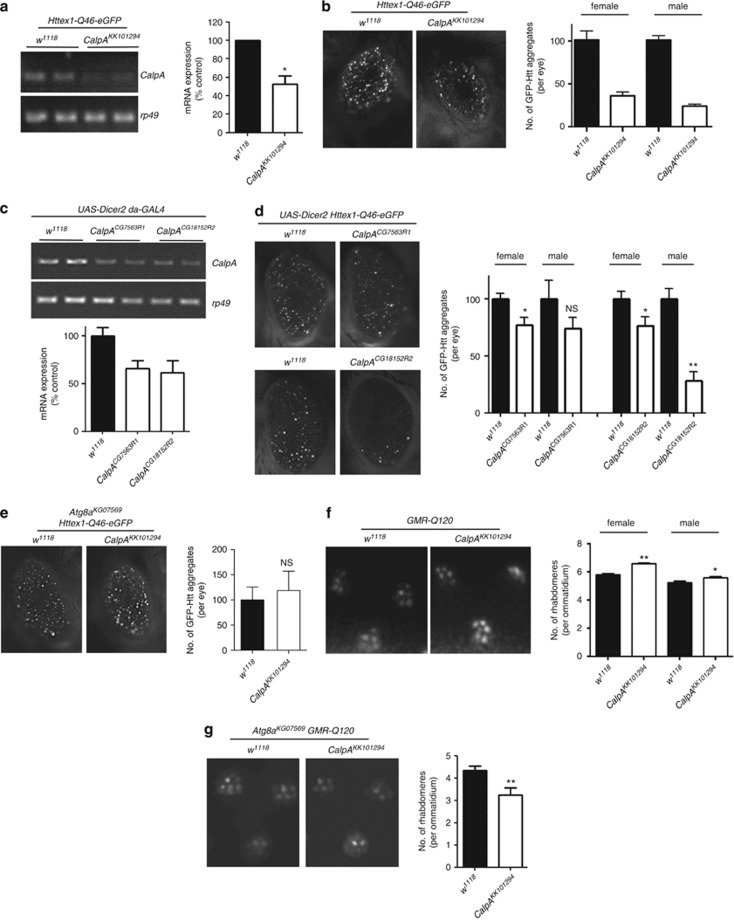
Calpain knockdown reduces Htt aggregation and protects against toxicity in *Drosophila*. (**a**) *CalpA* mRNA levels are decreased in heads of Httex1-Q46-eGFP flies expressing *CalpA*^*KK101294*^ *UAS-RNA* (*CalpA*^*KK*^), compared with control. Rp49 was used as an internal mRNA control. Genotypes: *w; GMR-GAL4/+ UAS-Httex1-Q46-eGFP/+* for control (*w*^*1118*^) and *w; GMR-GAL4/CalpA*^*KK101294*^*; UAS-Httex1-Q46-eGFP/+*. (**b**) Downregulation of *CalpA* in the eye reduces the number of aggregates in male and female *Httex1-Q46-eGFP* flies. (**c**) *CalpA* mRNA levels in adult flies are decreased using *CalpA*^*CG7563R1*^ *or CalpA*^*CG18152R2*^ *UAS-RNAi* lines expressed using *da-GAL4*, compared with control. Rp49 was used as an internal mRNA control. Genotypes: *UAS-dicer2; da-GAL4/+* for control and *UAS-dicer2; CalpA CG7563R1; da-GAL4/+* or *UAS-dicer2; da-GAL4/ CalpA CG18152R2* (**d**) Downregulation of *CalpA* in the eye using *UAS-Dicer2* and NIG *UAS-RNAi* lines reduces the number of aggregates in female and male *Httex1-Q46-eGFP* flies. Genotypes: *UAS-dicer2; GMR-GAL4/+ UAS-Httex1-Q46-eGFP/+* for control and *UAS-dicer2; GMR-GAL4/CalpA*^*CG7563R1*^*; UAS-Httex1-Q46-eGFP/+* or *UAS-Dicer2; GMR-GAL4/+ UAS-Httex1-Q46-eGFP/CalpA*^*CG18152R2*^. (**e**) Loss of *Atg8a* abolishes the rescue of aggregate formation by downregulation of *CalpA* in *Httex1-Q46-eGFP* flies. Genotypes: *Atg8a*^*KG07569*^*/Y; GMR-GAL4/+ UAS-Httex1-Q46-eGFP/+* for control and *Atg8a*^*KG07569*^*/Y; GMR-GAL4/ CalpA*^*KK101294*^*; UAS-Httex1-Q46-eGFP/+.* (**f**) *CalpA* knockdown increases the number of rhabdomeres per ommatidium in flies constitutively expressing mutant huntingtin in the eye (*GMR-Q120*). Genotypes: *w; elav-GAL4/+ GMR-Q120/+* for control and *w; elav-GAL4/CalpA*^*KK101294*^*; GMR-Q120/+*. (**g**) The protective effect on toxicity is lost in male flies lacking *Atg8a.* Genotypes: *Atg8a*^*KG07659*^*/Y; elav-GAL4/+ GMR-Q120/+* for control and *Atg8a*^*KG07659*^*/Y; elav-GAL4/CalpA*^*KK101294*^*; GMR-Q120/+* All graphs show mean±S.E.M. Comparisons with controls were performed using Student's *t*-tests: NS, not significant; *P<0.05; **P<0.01; ***P<0.001

We have previously demonstrated that siRNA knockdown of calpain in mammalian cell culture results in an upregulation of autophagy and a decrease in mutant Htt aggregation and toxicity.^8^ In order to extend this finding *in vivo,* we investigated the effect of *CalpA* RNAi knockdown in *Drosophila* expressing green fluorescent protein (GFP)-tagged huntingtin exon-1 with an expanded polyglutamine repeat (Httex1-Q46-eGFP)^17^ in the eye using *GMR-GAL4*. Aged *GMR-GAL4* Httex1-Q46-eGFP flies form GFP-fluorescent huntingtin aggregates that can be easily counted. After confirming that *CalpA* mRNA levels were decreased by *CalpA* RNAi expression (*CalpA*^*KK101294*^ from the Vienna *Drosophila* Resource Center, expressed in eyes using *GMR-GAL4*) in heads of flies expressing Httex1-Q46-GFP (Figure 1a), we observed that decreased *CalpA* levels resulted in a clear reduction in the number of Httex1-Q46-GFP aggregates in the eye, relative to the control flies (Figure 1b). This reduction in aggregates is likely to reflect a decrease in the soluble and oligomeric forms of mutant huntingtin as well as the large aggregates, as these forms have all been demonstrated to be altered by autophagy induction.^6^ To confirm the phenotypes observed using the *CalpA*^*KK101294*^ RNAi line, we used two insertions of a second UAS RNAi construct (*CalpA*^*CG7563R1*^ and *CalpA*^*CG18152R2*^), from the Japanese National Institute of Genetics (NIG-Fly Stock Center), which had no sequence overlap with *CalpA*^*KK101294*^. Both these lines were effective in decreasing *CalpA* mRNA levels (Figure 1c), and expression in eyes using *GMR-GAL4* resulted in a decreased number of Httex1-Q46-GFP aggregates (Figure 1d), similar to *CalpA*^*KK101294*^. To test the contribution of autophagy to this decrease in aggregate number, we assessed the effects of calpain knockdown in flies with a loss-of-function allele for the key autophagy gene *Atg8a*.^18^ In *Atg8a* mutant flies, reduction of *CalpA* levels was unable to reduce Htt aggregate number (Figure 1e).

To establish whether this reduction in aggregate number correlated with an increase in cell survival, we used the pseudopupil technique.^19^ Neurodegeneration in the *Drosophila* eye can be observed as a decrease in the number of visible rhabdomeres within each of the ommatidium that make up the compound eye. Flies expressing the first exon of huntington with an expanded polyglutamine repeat region of 120 in the eye (GMR-Q120) show a progressive decrease in the number of visible rhabdomeres per ommatidium,^20^ a phenotype that was partially rescued upon *CalpA* downregulation (Figure 1f). As was seen for the effect on aggregation, this decreased toxicity was not observed in GMR-Q120 flies in a mutant *Atg8a* background (Figure 1g). In fact, decreasing *CalpA* in these autophagy-defective flies increased the toxicity of mutant Htt, further demonstrating a role for autophagy in the protective effect of calpain inhibition.

### CalpA downregulation is also protective in *Drosophila* expressing other proteins associated with neurodegeneration

**Figure 2:**
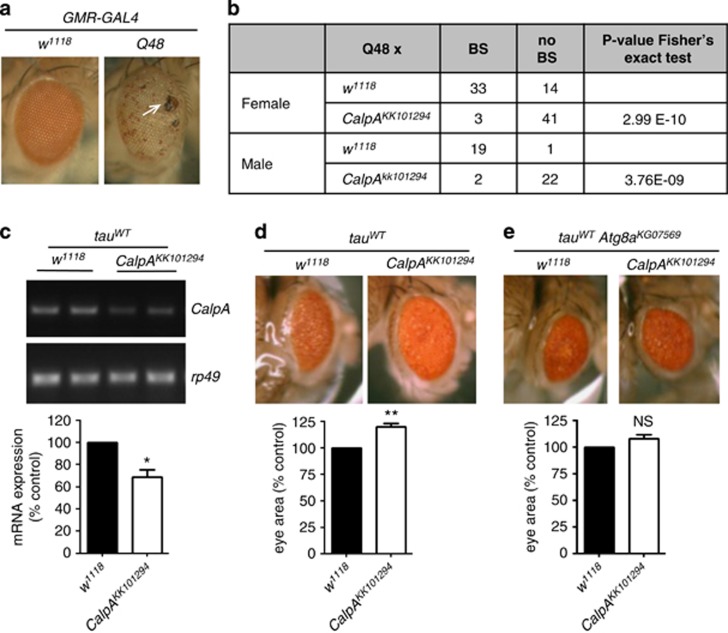
Downregulation of *CalpA* ameliorates *Q48* and *tau*^*WT*^ eye phenotypes. (**a**) Expression of a stretch of 48 glutamines in the eye (Q48) causes severe loss of pigmentation and presence of blacknecrotic-like spots (labelled with a white arrow) compared with WT flies. Genotypes: *w; GMR-GAL4/+* for control (*w*^*1118*^) flies and *w; GMR-GAL4/+ UAS-Q48.myc.flag/+* for Q48. (**b**) Downregulation of *CalpA* using *CalpA*^*KK101294*^ reduces the number of black necrotic-like spots of Q48 flies. Fisher's exact test was applied for statistical comparisons between the control and test genotypes. BS= black necrotic-like spots; no BS= absence of black necrotic-like spots. (**c**) *CalpA* mRNA levels are decreased in the eye of flies expressing both *tau*^*WT*^ and *CalpA*^*KK101294*^ compared with controls expressing *tau*^*WT*^ alone. *Rp49* was used as an internal mRNA control. Genotypes: *w; GMR-GAL4/+ UAS-tau*^*WT*^*/+* and *w; GMR-GAL4/ CalpA*^*KK101294*^*; UAS-tau*^*WT*^*/+*. (**d**) Downregulation of *CalpA* using *CalpA*^*KK101294*^ increases the eye area of flies expressing tau^WT^. Genotypes: *Atg8a*^*KG07659*^*/Y; GMR-GAL4/+ UAS-tau*^*WT*^*/+* for control and *Atg8a*^*KG07659*^*/Y; GMR-GAL4/CalpA*^*KK101294*^*; UAS-tau*^*WT*^*/+*. (**e**) Loss of *Atg8a* abolishes the protective effect of *CalpA* downregulation on *tau*^*WT*^. All graphs show mean±S.E.M.; comparisons with controls were performed using paired Student's *t*-tests. NS, not significant; *P<0.05; **P<0.01

To test these effects in a related model with a different readout, we expressed an epitope-tagged naked stretch of 48 polyglutamines (Q48) in the fly eye using the GMR-GAL4 driver.^21^ Expression of this protein causes severe eye degeneration with loss of pigmentation in the eye and formation of black necrotic-like spots (Figure 2a). Downregulation of *CalpA* in Q48 flies led to a significant reduction in the number of flies with black necrotic-like spots in the eye, in both males and females (Figure 2b), demonstrating a protective effect of reduction of *CalpA* levels against neurodegeneration resulting from expression of pure polyglutamine proteins.

The protective effect of *CalpA* downregulation has also been previously shown in flies overexpressing tau,^22^ which aggregates intracellularly in Alzheimer's disease and is mutated in forms of frontotemporal dementia. However, this study argued that the protection was via interference of tau proteolysis by calpains, and the mutant alleles used do not disrupt the calpain coding regions (www.flybase.org) and only reduced calpain activity by 5–10%. We were able to confirm that calpain knockdown using the *CalpA*^*KK101294*^ line (Figure 2c) protected against tau toxicity, as measured by the eye area of flies expressing wild-type (WT) tau^23^ (Figure 2d). As we observed for the polyglutamine proteins, protection due to decreased *CalpA* levels required functional autophagy as the protective effect was lost in flies on an *Atg8a* loss-of-function background (Figure 2e). Decreasing *CalpA* levels is therefore protective against diverse proteins associated with neurodegenerative disorders and requires functional autophagy.

### CAST overexpression increases autophagy and reduces Htt levels in a mouse model of HD

**Figure 3:**
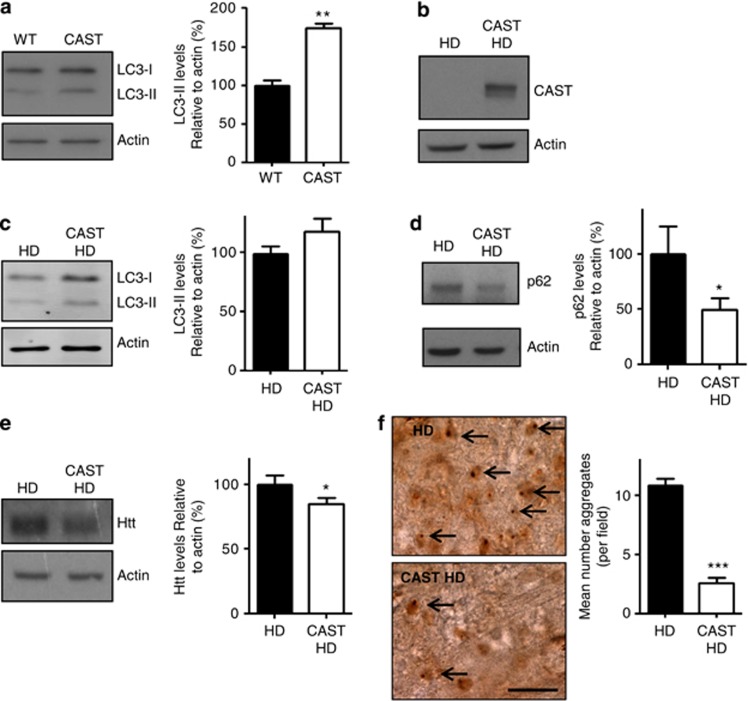
Calpastatin overexpression enhances autophagy in mice and reduces Htt levels. (**a**) LC3-II levels in brain lysates from mice overexpressing calpastatin (CAST, white bar) and littermate controls (WT, black bar) were assessed by western blotting, blots were also probed for actin as a loading control. Graph shows densitometric analysis of LC3-II-levels relative to actin. Control condition is set to 100%. (**P<0.005, *t*-test). (**b**) CAST overexpression in double-transgenic calpastin, N171-82Q mice (CAST HD) relative to single-transgenic N171-82Q mice (HD). (**c**) LC3-II levels in brain lysates from mice overexpressing calpastatin (CAST HD, white bar *n*=9) on an N171-82Q background (HD, black bar *n*=8). Graph shows densitometric analysis of LC3-II-levels relative to actin. (**d**) Levels of p62 in brain lysates from HD mice (HD, black bar *n*=8) and HD mice also overexpressing (CAST HD, white bar *n*=9). Graph shows densitometric analysis of p62 levels relative to actin (*P<0.05, *t*-test). (**e**) Soluble huntingtin (Htt) levels in the cytoplasmic fraction of brain lysates from HD mice overexpressing CAST in comparison to control, HD mice. Graph shows densitometric analysis of Htt levels relative to actin (HD *n*=8, CAST HD *n*=9) (*P< 0.05, *t*-test). (**f**) The number of huntingtin-positive aggregates in the motor cortex of N171-82Q mice was reduced by the overexpression of CAST. Examples of aggregates are highlighted by arrows. Scale bar represents 50 *μ*M and is valid for both images. Mean number of aggregates per field was quantified in three mice per group and the quantification is shown in the graph (***P<0.001, *t*-test). All graphs represent mean and error bars represent S.E.M.

To test these phenomena in a mammalian system, we used mice overexpressing CAST, which inhibits calpain activity.^24^ CAST expression was driven by a thymocyte differentiation antigen 1.1 (Thy1.1)-expression cassette and was therefore limited to the brain. Western blotting in brain tissue isolated from transgenic mice overexpressing CAST demonstrated increased levels of LC3-II, a modified form of LC3 known to associate with autophagosomes and the only known marker for autophagosome number (Figure 3a). This increase in LC3-II is therefore compatible with an increase in autophagy in CAST overexpressing mice. Although an increase in LC3-II might, in principle, arise from enhanced autophagosome formation or impaired autophagosome degradation, the latter is unlikely in view of our previous data in cells showing that CAST increased autophagosome formation^8^ and the dependence of *Drosophila* calpain knockdown phenotypes on functional Atg8a, described above. The HD model mice (N171-82Q) overexpress an exon-1 fragment of the huntingtin protein with an expanded polyglutamine region of 82Q. They have a progressive neurodegenerative disease and show signs of motor impairment on rotarod and grip-strength tests, as well as increased tremors and intracellular huntingin aggregates. In these mice, the transgene is driven by the mouse prion protein promoter resulting in expression predominantly in the brain (as with the CAST overexpressing mice), unlike the R6/2 mice where the transgene is expressed widely in the peripheral tissues such as muscle.^25^

To investigate whether calpain inhibition confers protection against neurodegeneration, we expressed CAST in N171-82Q mice (Figure 3b). Mice expressing the expanded polyglutamine transgene (HD) were compared with the littermates expressing both mutant huntingtin and CAST (CAST HD). Western blot analysis of the brains of HD mice demonstrated that overexpression of CAST resulted in an increase in LC3-II levels (Figure 3c); however, this increase was relatively small and did not reach statistical significance. This may be due to the presence of mutant Htt interfering with the upregulation of autophagy by CAST, but may also be due to lack of sensitivity of the LC3-II western blot autophagy assay in mouse brain, especially as this assay *in vivo* only allows one a snapshot of the autophagosome load without any direct inference of flux, as one cannot easily block flux *in vivo* with lysosomal inhibitors. However, an increase in functional autophagy in these CAST HD mice was consistent with the observed decrease in the autophagy adaptor protein p62, which is also degraded by autophagy (Figure 3d). We have previously demonstrated that mutant Htt is also degraded by autophagy,^2^ and in agreement with an upregulation of autophagy in these mice, we saw a decrease in the levels of SDS-soluble mutant Htt in the cytoplasmic fractions of brain lysates from CAST transgenic mice (Figure 3e). We also saw a dramatic decrease in the number of Htt-positive aggregates in the motor cortex of HD mice when they also expressed CAST (Figure 3f).

### CAST overexpression is protective in a mouse model of HD

**Figure 4:**
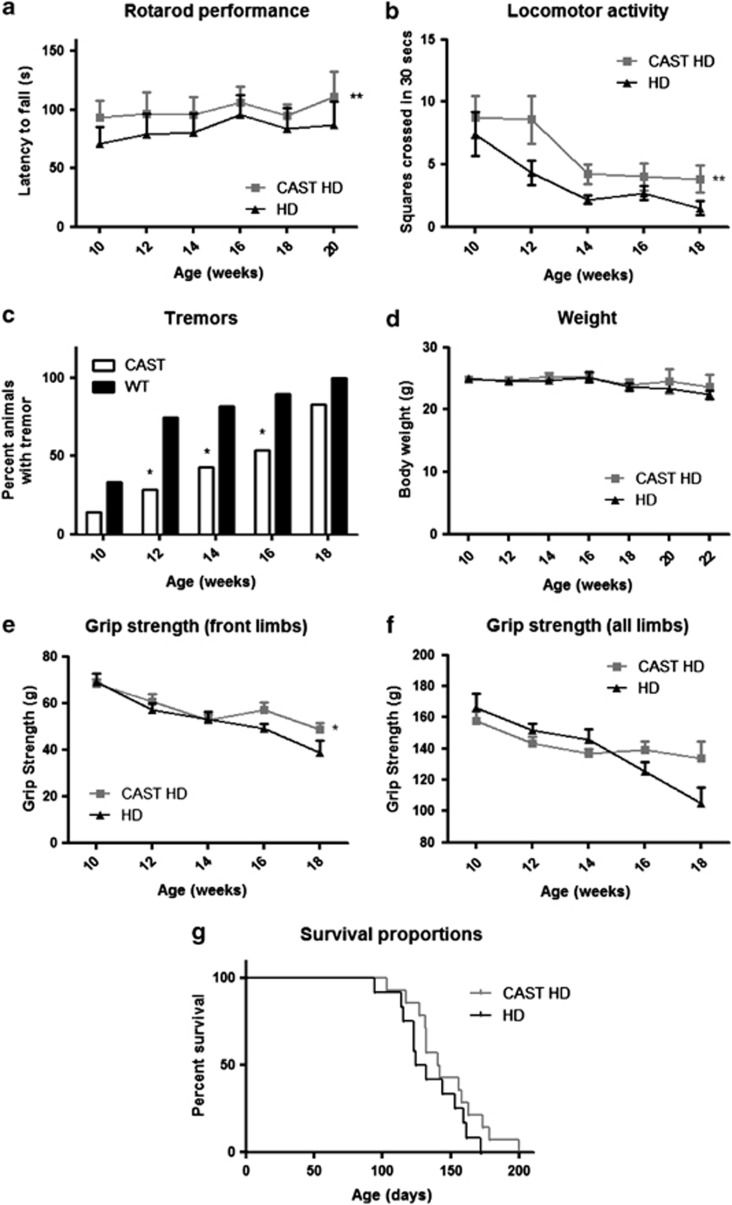
CAST overexpression improves motor performance and the severity of tremors in a transgenic mouse model of HD. (**a**) Calpastatin overexpression (CAST HD, grey line) improves best rotarod performance over a trial of three tests compared with control N171-82Q mice (HD, black line). Overall effect obtained from all age points, **P<0.005 (two-way ANOVA). (**b**) CAST overexpression in N171-82Q mice (CAST HD, grey line) improves locomotor activity measured by open field test in comparison to control group (HD, black line). Overall effect from all the age points was **P<0.01 (two-way ANOVA). (**c**) The number of mice with tremors was significantly improved by CAST overexpression (CAST HD, white bars) in N171-82Q mice at 12 weeks (*P=0.0112), 14 weeks (*P=0.0286) and 16 weeks of age (*P=0.0369) in comparison to the control group (HD, black bars) (Mann–Whitney *U*-test). (**d**) Body weight is not changed in 171-82Q transgenic mice when they also overexpress CAST. (**e**) Forelimb grip strength in N171-82Q mice (black line) compared with mice also overexpressing CAST (grey line). Overall effect from all age the points, (*P=0.0397) (two-way ANOVA) (**f**) Grip strength of all limbs in N171-82Q mice (black line) compared with mice also overexpressing CAST (grey line) (*P*=NS, two-way ANOVA). (**g**) The median survival age of CAST-overexpressing mice (CAST HD) is compared with the control group (HD) (128 days *versus* 141 days, *P*=0.08, Mantel–Cox test, 1 tailed). All graphs represent mean and error bars represent S.E.M.

In addition to the cellular effects of CAST overexpression in HD mice, we sought to investigate if disease progression was also altered in these animals. Behavioral tests were carried out on both HD and CAST HD mice beginning at 10 weeks of age, around the time that the first signs of disease manifest, and then repeated every fortnight. Motor coordination was assessed using rotarod performance. Throughout the trial period CAST-overexpressing mice displayed a greater ability in the rotarod test than the single-transgenic HD littermates (Figure 4a and Supplementary Figure 1a). As another measure of locomotor activity, the open field test was used. CAST HD mice were seen to have increased locomotor activity at all stages tested relative to HD mice (Figure 4b and Supplementary Figure 1b). A reliable phenotype seen in the N171-82Q mice is the onset of tremors from an early age. Overexpression of CAST delayed the age of onset of tremors in the HD mice (Figure 4c). CAST overexpression did not influence the weight of the HD mice (Figure 4d and Supplementary Figure 1c). Grip strength is also decreased in HD mice relative to control mice (Supplementary Figures 1d and e), and there was some protection by overexpression of CAST at later stages of disease (Figures 4e and f). HD mice have a decreased lifespan. According to our Project Licence, animals were killed when they showed medium severity symptoms, identified by signs such a weight loss of 20%, continuous tremor, hunching or subdued behavior when provoked. Although this compromises accurate lifespan assessments, CAST HD mice had median survival of 141 days compared with 128 days in single-transgenic HD mice (Figure 4g; *P*=0.08).

### Long-term overexpression of CAST in the brain is not obviously deleterious

**Figure 5:**
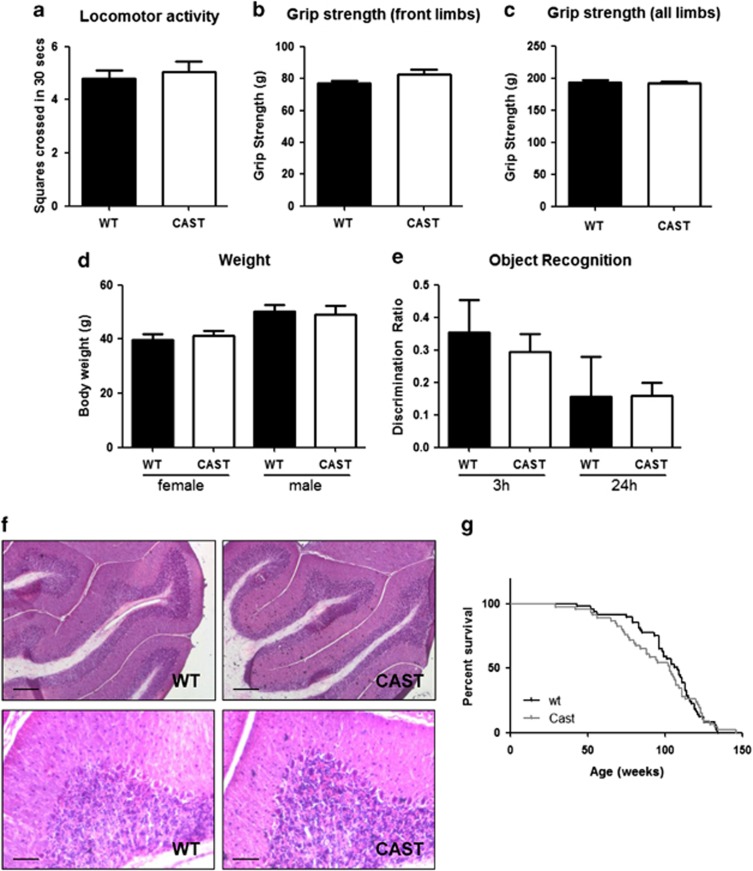
Calpastatin overexpression does not cause an overt phenotype in aged mice. (**a**) In 1.5-year-old mice calpastatin overexpression (CAST) did not alter locomotor activity, as measured by open field test, in comparison to control group (WT). (**b**) Forelimb grip strength in wild-type littermate controls (WT) compared with mice overexpressing CAST. (**c**) Grip strength of all limbs in WT control mice (black bar) compared with mice overexpressing CAST. (**d**) Body weight is not changed in CAST- transgenic mice (CAST) compared with the littermate controls (WT). Males were slightly heavier that females in both genotypes. (**e**) Mice did not show cognitive impairment based on spontaneous novel object recognition testing. Discrimination ratio plotted on the graphs represents the preference showed by the mice for investigating the novel object during the ‘Choice' phase, calculated as (time spent exploring the novel object−time spent exploring the sample object)/(total time spent exploring). (**f**) Hemotoxylin and eosin staining of CAST transgenic and control (WT) mice did not demonstrate any changes in morphology. Examples of staining in the cerebellum are shown at low magnification (top images, scale bar represents 200 *μ*m) and higher magnification (lower images, scale bar represents 50 *μ*m). (**g**) Survival was not altered in mice overexpressing CAST compared with the littermate controls (WT) (median lifespan; CAST 103 weeks, *n*=46, WT, 108 weeks, *n*=49, *P*=NS, Mantel–Cox test). In all graphs, white bars represent CAST- transgenic mice and black bars represent WT, littermate controls, error bars represent S.E.M.

The data described above suggest that decreased calpain activity can protect against neurodegeneration by enhancing the clearance of mutant huntingtin via autophagy. The ultimate aim of work such as this study is to identify potential therapeutic targets of relevance for HD. Such treatments, once developed, would have to be tolerated long-term by patients without any deleterious effects. We therefore sought to establish whether long-term inhibition of calpain activity in the brain and therefore long-term upregulation of autophagy resulted in any neurological consequences in mice. SHIRPA (SmithKline Beecham, Harwell, Imperial College, Royal London Hospital, phenotype assessment)^26^ was used as a primary screen to assess 1.5-year-old CAST mice for behavioral alterations compared with the non-transgenic littermate controls. SHIRPA is a phenotype screen that can identify broad neurological or muscular dysfunction. The test includes assessment of body position, limb tone, spontaneous activity, coordination and tail elevation as part of 30 semiquantitatively measured endophenotypes. This comprehensive assessment of phenotypes can reflect potential deficits in a variety of functions ranging from autonomic to neuropsychiatric and moto-sensory capabilities. A total of 22 non-transgenic mice were assessed (11 female and 11 male) and 19 CAST mice (11 female, 8 male), and no abnormal signs were observed in CAST mice (Supplementary Table 1). In addition, no change was seen in the locomotor activity of the mice as assessed by open field testing (Figure 5a), the grip strength of CAST-overexpressing mice was not altered relative to controls (Figures 5b and c) and there was no change in weight (Figure 5d). In order to investigate the cognitive ability of these mice, spontaneous novel object recognition tests were employed. Mice presented with two objects, one familiar and one novel, will spend longer investigating the novel object. The ratio of time spent investigating the novel and familiar object can be used as a readout for the recognition of the object by the mouse and therefore memory and cognitive ability. No difference was observed between CAST and WT mice in this test at a 3 or 24 h delay (Figure 5e). There was also no difference in the amount of exploration during the sample or choice phase of the CAST and WT mice. One CAST mouse was removed from the 24 h delay analysis because of low (<2 s) exploration during the ‘Sample' phase.

Gross brain morphology, assessed by hematoxylin and eosin staining and microscopy revealed no major alterations (an example is shown in Figure 5f). Finally, survival of the CAST mice was assessed relative to control mice. These transgenic mice are on a C57BL/6 background and as such are prone to developing a skin condition, ulcerative dermatitis.^27^ Although this is not life-threatening, any mice developing this condition were culled to reduce discomfort and excluded from the survival study. No change in frequency was observed in the frequency of the dermatitis in CAST, compared with the littermate control mice. CAST overexpression did not significantly change the lifespan of the mice (median survival CAST 103 weeks *n*=46, WT 108 weeks *n*=49, not significant; no difference was seen in survival when comparing males and females separately, data not shown). Although our data suggest that CAST overexpression/calpain inhibition in the brain is well tolerated, further models with whole-body CAST overexpression will be required to test if this is similarly well tolerated in all organs.

## Discussion

In this study we demonstrate that reducing *CalpA* levels in *Drosophila* results in protection against phenotypes resulting from Htt overexpression in a manner dependent on functional autophagy. This protective effect is also seen in mammalian models of HD as overexpression of the endogenous calpain inhibitor, CAST, has a beneficial effect in mice overexpressing Httex-1 with an expanded polyglutamine repeat. As this short Htt fragment does not contain any calpain-binding sites and is the same in length as the short, toxic fragments that have been implicated in the toxicity of huntingtin,^16^ this protective effect cannot be due to decreased calpain cleavage of mutant Htt.

We have observed that overexpression of CAST in mice results in a large decrease in the number of aggregates present in the brain and a more modest decrease in levels of SDS-soluble Htt. However, the preferred substrates for autophagic degradation are likely to be the SDS-soluble oligomeric forms. The large aggregates seen by light microscopy in HD are never membrane bound and are much larger than the typical 1-micron diameter of an autophagosome. Decreasing oligomer levels will impact on both the SDS-soluble pool and the numbers of aggregates, as the oligomers are the precursors of the aggregates. As the SDS-soluble pool likely has a greater proportion of monomers than oligomers, the change in this pool with autophagy induction would be expected to be rather modest. Furthermore, the ratio of aggregates to soluble protein is controlled by the aggregation kinetics of the mutant protein. Aggregate formation correlates with the total concentration of the mutant protein.^28^ Therefore as the total protein concentration (soluble and aggregated) decreases the number of aggregates will decrease until the concentration is below the threshold required for protein aggregation. This may be an additional factor that explains why the aggregate number changes more dramatically than the SDS-soluble mutant huntingtin.

Although the loss of autophagy in flies abrogates the protective effect of decreased *CalpA* levels, it is still possible that factors other than autophagy upregulation contribute to the protective effect seen in mouse HD models, as alterations in calpain activity have been associated with HD in a variety of ways. Calpain activation has been demonstrated in the brains of HD patients in both the putamen^29^ and caudate.^14^ This activation is not seen either in age-matched controls or in the regions of the brain that are less vulnerable in HD such as the frontal cortex.^29^ It may be explained by an increase in neuronal calcium levels caused by excitotoxicity, which has been implicated in HD via a number of mechanisms.^30^

Calpain dysregulation and a protective effect of calpain inhibition have also been suggested in a variety of other neurodegenerative diseases. In Alzheimer's disease calpains have been shown to be aberrantly activated^29^ and have been implicated in the processing of amyloid precursor protein^31^ and tau.^32,33^ Consistent with this, a decrease in CAST in brains of Alzheimer's disease patients has been reported^24^ and overexpression of CAST is protective in mice injected with kainic acid, a model of excitotoxicity associated with Alzheimer's disease.^24^ Similarly, marked depletion of CAST has been seen in mice expressing P301L-mutant tau and overexpression of CAST prevents a diverse range of tauopathy-associated phenotypes seen in these mice, including delaying disease onset by 3 months.^34^ Overexpression of CAST is also protective in mouse models of Parkinson's disease. Calpains have been suggested to cleave *α*-synuclein and enhance its aggregation properties.^35^ Transgenic mice overexpressing A30P mutant *α*-synuclein along with CAST showed a decreased number of *α*-synuclein positive aggregates and decreased neuropathology compared with mice overexpressing A30P mutant *α*-synuclein alone.^36^ Although the mechanism for the protective effect of CAST in these cases has been suggested to be decrease the cleavage of the relevant protein, these proteins have also been demonstrated to be substrates for autophagic clearance.^3,37^ This hints that autophagy upregulation could contribute to the protective mechanisms, as we have seen in HD in this study. This has not yet been formally tested, but could suggest a double-protective effect for decreasing calpain activity in a range of neurodegenerative diseases. It must also be considered that the protective effect seen in the HD mouse model in this study is relatively mild, the potential double-protective effect would not apply to this model expressing only exon-1 Htt, and the speed of disease progression in this model relative to the very slow onset in humans may reduce the window of opportunity for reducing protein levels and relieving disease signs. Investigating the protective effect of calpain inhibition in other HD models (e.g., those expressing longer Htt variants) may therefore be of value. Despite this, when considered with the observations in this study that decreasing calpain activity over long periods of time does not have obvious deleterious effects in mice, calpain inhibition could be a promising therapeutic strategy to explore in these diseases.

## Materials and Methods

### Fly stocks

For knockdown of *CalpA* the *UAS-RNAi* line KK101294 (*CalpA*^*KK101294*^), the *w*^*1118*^ control stock (60100) from Vienna *Drosophila* Resource Center (VDRC, http://stockcenter.vdrc.at/control/main),^38^ the *UAS-RNAi* lines *CG7563R1* or *CG18152R2* (*CalpA*^*CG7563R1*^ or *CalpA*^*CG18152R2*^), or the *w*^*1118*^ control from the Japanese National Institute of Genetics (NIG-Fly, http://www.shigen.nig.ac.jp/fly/nigfly/) were used. The KK construct targets *CalpA* exons between genomic coordinates 15314791 and 15315502 (release 5.57), and both NIG-Fly lines target *CalpA* exons between genomic coordinates 15313689 and 15314791; note that *CG7563* and *CG18152* are both synonyms for *CalpA* (www.flybase.org). GAL4 drivers used were *P{GAL4-ninaE.GMR}12* (*GMR-GAL4*),^39^ *P{GAL4-da.G32}* (*da-GAL4*),^40^ *P{GAL4-elav.L}2* (*elav-GAL4*)^41^ and *P{GawB}elav*^*C155*^ (*elav-GAL4*^*c155*^).^42^ Other fly stocks used were *UAS-tau*^*WT*^ (*tau*^*WT*^, gift of Mel Feany),^23^ *UAS-Httex1Q46-GFP* (*Httex1-Q46-eGFP*, gift of Sheng Zhang),^17^ *P{GMR-HTT.Q120}4.62* (*GMR-Q120*, gift of G Jackson),^20^ *P{UAS-Q48.myc.flag}31* (*Q48*, gift of Lawrence Marsh),^21^ *P{SuPor-P }Atg8a*^*KG07569*^ (*Atg8a*^*KG07569*^, Bloomington Stock Center),^43^ *P{UAS-Dicer2, w[+]}* (*UAS-Dicer2*, VDRC).

### Aggregate counting in Huntingtin-mutant flies

Flies expressing GFP-tagged expanded huntingtin exon 1 in the eye (*Httex1-Q46-eGFP*^17^) were crossed to *UAS-RNAi* line *CalpA*^*KK101294*^ or to *w*^*1118*^ control flies (in the presence or absence of the loss-of-function allele *Atg8a*^*KG07569*^). For the *UAS-RNAi* lines from the NIG-Fly stock, virgins *UAS-dicer2; CalpA*^*CG7563R1*^ or *UAS-dicer2; + CalpA*^*CG18152R2*^ were crossed to *w; GMR-GAL4; UAS-Httex1-Q46-eGFP* males. The progeny were collected and analyzed 15 days post eclosion or 35 days post eclosion in the case of NIG-Fly *UAS-RNAi* lines, with age-matched controls imaged concurrently. The crosses were performed at least four times with at least four flies analyzed per cross. All crosses were performed and maintained at 25 °C. Images were acquired using a fluorescence stereomicroscope (Leica MZ16 F, Milton Keynes, UK). GFP intensity or the number of aggregates per eye were measured using ImageJ (U.S. National Institutes of Health, Bethesda, MD, USA; http://ImageJ.nih.gov), data were exported to GraphPad Prism 5 (GraphPad Software, San Diego, CA, USA) and statistical significance was determined using two-tailed unpaired Student's *t*-tests for the experiment with *CalpA*^*KK101294*^ or one-tailed unpaired Student's *t*-tests for *CalpA*^*CG7563R1*^ and *CalpA*^*CG18152R2*^.

### Pseudopupil analysis on mutant huntingtin flies

Analysis was performed as previously described.^19^ In brief, a stock line constitutively expressing in the eye mutant huntingtin exon 1 (*GMR-Q120*)^20^ with either *CalpA*^*KK101294*^ or its background control was crossed to the neuronal driver *elav-GAL4*^*C155*^^42^ at 25° C. For analysis using the *Atg8a*^*KG07659*^ loss-of-function line, *Atg8a*^*KG07659*^ virgins carrying either *CalpA*^*KK101294*^ or its background control were crossed to *w; elav-GAL4; GMR-Q120* males. The number of rhabdomeres per ommatidium was scored in progeny of the above crosses at 3 days post eclosion. Statistical analysis was performed using Student's *t*-tests on data from at least five independent experiments, each based on ~10 individuals for each genotype, scoring 15 ommatidia per eye.

### *Tau*^*WT*^ eye area analysis

Virgins of *w; GMR-GAL4;UAS-tau*^*WT*^ were crossed either with *CalpA*^*KK101294*^ or with *w*^*1118*^ control males. For analysis using the *Atg8a*^*KG07659*^ loss-of-function line, *Atg8a*^*KG07659*^ virgins carrying either *CalpA*^*KK101294*^ or its background control were crossed with *w; GMR-GAL4;UAS-tau*^*WT*^ males. The progeny were collected and analyzed 3 days post eclosion, with age-matched controls imaged concurrently. All the crosses were performed five times at 25 °C with at least six male flies analyzed per cross. Images were acquired using a × 7.5 light microscope (SMZ-100, Nikon, Kingston upon Thames, UK). Eye area was measured using ImageJ blind to genotype. Data were exported to GraphPad Prism 5 (GraphPad Software Inc.) for statistical analysis. Statistical significance was determined using Student's *t*- tests with five independent experiments.

### Semiquantitative PCR

Total RNA was purified from >40 fly heads (GMR-GAL4 crosses) or from 3 flies (da-GAL4 crosses) collected in TRIzol reagent (Invitrogen, Paisley, UK). cDNA was produced using SuperScript III First Strand kit (Invitrogen), 50 *μ*M oligo-dT primer and 2 *μ*g DNase-treated RNA. Amplification of Rp49 mRNA was used to control for cDNA concentration in the polymerase chain reaction (PCR). Primers used were: Rp49-F: 5′-CCGACCACGTTACAAGAACTCTC-3′, Rp49-R: 5′-CGCTTCAAGGGACAGTATCTGA-3′, CalpA-F: 5′-TCCGAGGTGCAGGACTATGA-3′ and CalpA-R: 5′-GTTCTTCTCCGTGGAGTGCA-3′. PCR conditions were 95 °C for 30 s; 60 °C 30 s; and 72 °C for 1 min repeated for 20 cycles for Rp49 and 30 cycles for CalpA. Samples from at least three independent crosses for each genotype were analyzed. PCR products were visualized with ethidium bromide on a gel, and band intensities quantified using ImageJ.

### Characterisation of *CalpA* KK-RNAi line *CalpA*^*KK101294*^

We verified that the *CalpA*^*KK101294*^*UAS-RNAi* line was not inserted at the landing site that affected the *tio* gene^44^ by crossing virgins of *elav-GAL4*^*C155*^^42^ with either *CalpA*^*KK101294*^ or *w*^*1118*^ (60100 stock, VDRC Stock Center, http://stockcenter.vdrc.at/control/main) males. The progeny of both crosses had normally inflated wings (data not shown), suggesting that they harbour the targeted insertion at the anticipated genomic site.^44^

### Mouse models

We used HD-N171-82Q mice backcrossed on a C57BL/6J background for more than ten generations. These mice carry an N-terminal fragment expressing the first 171 amino acids of human huntingtin with 82 glutamine repeats under the mouse prion promoter.^45^ CAST- transgenic mice express CAST throughout the cortex and hippocampus under the control of the Thy 1.1 promoter.^24^ Mice were genotyped by PCR with DNA extracted from ear-clips at 3 weeks of age (primer sequences available on request). To generate the double-transgenic mice, we crossed N171-82Q heterozygous males with Thy-Cast heterozygous females. All studies and procedures were performed under the jurisdiction of appropriate Home Office Project and Personal animal licenses and with local Ethics Committee approval.

The following signs were used as humane end points for the mice, which resulted in euthanasia: marked loss of appetite and fluid intake, staring coat, hunched posture and subdued behavior, or 20% weight loss over a period of <3 days.

### Behavioral analysis

Animals were housed together in groups of mixed genotypes, and experienced observers were blind to the genetic status during testing. Mice were carefully monitored daily, and weighed every week. A minimum of eight males per genotype and line were analyzed for specific tests. They were housed under conventional conditions in individually ventilated cages with food and water *ad libitum*.

We assessed the motor performance at 10, 12, and 14 weeks of age with a rotarod apparatus (Accelerating Model, Ugo Basile, Biological Research Apparatus, Varese, Italy). The mice were given training sessions for two consecutive days to acclimatize them to the apparatus and on the third day, the definitive testing took place. On the first training day, the mice had three trials at a constant speed of 4  r.p.m. In each trial, the animals were put on the rotarod for a maximum of 300 s. On the second day, the training took place at a constant speed of 10 r.p.m. (three trials). Day 3 was the testing day, where the speed was accelerated from 3 to 30  r.p.m. in 300 s. A minimum of 10 min break was given between each trial. The latency to fall was taken as the maximum value reached over the three trials.

Grip strength and tremor monitoring was also performed at 10, 12 and 14 weeks of age. Grip strength was monitored quantitatively by using a grip strength meter (Bioseb, Vitrolles, France). The mice were held above the apparatus grid with their front paws (forelimb grip) or all four paws (all limb grip) grasping the grid, then pulled back by the tail following the axle of the sensor, horizontally and steadily, until they released the grid. The apparatus was used in the peak mode, the recorded value corresponding to the maximum force produced by the animal. Forelimb grip strength as well as all limb grip strength was measured three times and the average value was taken. For tremors, the mice were placed on a grid in a clear perspex cylinder. We recorded tremor for 2 min and scored the mice as follows: 0, none; 1, tremor.

Aged-CAST mice were also tested for rotarod and grip strength performance. SHIRPA assessment was completed as previously described.^26^

### Spontaneous novel object recognition

Recognition memory was assessed at 19 months of age, using a spontaneous novel object recognition (SOR) paradigm. A total of 18 CAST mice and 8 non-transgenic littermate controls, were housed in groups of 2–3 on a 12-h light cycle (lights on 19:00–07:00) and testing was performed during the dark phase of the cycle. Mice were provided with *ad libitum* access to water, but food was restricted beginning 5 days before the start of testing to maintain body weight to 85–90% their free-feeding weight. Mice were handled for 2 days before testing.

SOR testing took place in a Y-shaped apparatus (previously described in^46^), made of homogenous opaque white perspex. Walls were 30 cm high, and each arm was 16 cm in length and 8 cm wide. A digital video camera was mounted above the Y-maze to record the trials. One arm was used as the start arm, and the other two arms were used to present the testing stimuli (randomly shaped junk objects ~10 cm × 4 cm × 4 cm) secured to the floor of the maze with Blu-tack. All mice were habituated to the apparatus for three consecutive daily sessions in which they were placed in the empty apparatus for 5 min and allowed to freely explore.

Testing consisted of two phases. During the ‘Sample' phase, a mouse was shown two identical sample objects and allowed to explore for 5 min. The mouse was then removed from the maze and placed back in its homecage. After a pre-determined delay of 3 or 24 h, the mice were then placed back into the Y-maze for the ‘Choice' phase and shown one copy of the sample object and one novel object. All mice were tested with a 3 h delay and then 5 days later were tested with a 24 h delay. The maze and objects were wiped with a 50% ethanol solution between trials and the side of the maze in which the novel object was presented, was counterbalanced.

Exploration was defined as a mouse directing its nose to an object at a distance of 2 cm or less. Climbing on, sitting on or chewing the object was not included as exploration. The experimenter scored exploration using a computer program JWatcher_V1.0, written in Java (http://www.JWatcher.ucla.edu). The program had two keys corresponding to the two objects. Exploration was recorded by pressing the appropriate keys at the onset and offset of a bout of exploration.

For the ‘Choice' phase, discrimination ratios (*D*2) were calculated as the time spent exploring the novel object minus the time spent exploring the sample object divided by the total exploration time. Because of rodents' innate preference toward novelty, a *D*2>0, which suggests a preference toward exploring the novel object, is thought to reflect a memory for the sample object.

### Protein extraction and western blot analysis

Brains were frozen immediately after removal and stored at −80 °C for all possible genotypes. They were mechanically homogenized in lysis buffer containing 50 mm Tris, pH 7.4, 0.5% Triton X-100 and protease inhibitor cocktail. The homogenate was centrifuged at 13 400 *g* at 4 °C, the supernatant was removed and used for western blot. Cell pellets were lysed on ice in Laemmli buffer (6.5 mM Tris-HCl, pH 6.8, 2% sodium dodecyl sulphate (SDS), 5% *β*-mercaptoethanol, 10% glycerol, 0.01% Bromophenol blue and protease inhibitors) for 30 min. Proteins were separated on 12% SDS-polyacrylamide gels and transferred onto PVDF membranes (Hybond enhanced chemiluminescence (ECL) membrane, GE Healthcare, Little Chalfont, UK). Membranes were blocked by incubation in 5% dried milk in PBS and 0.1% Tween-20, pH 7.6. The primary antibodies used were anti-LC3 (1 : 10 000; Novus Biologicals, Cambridge, UK), anti-p62 (1 : 1000; BD, Oxford, UK) anti-actin (1 : 1000; Sigma, Dorset, UK). Horseradish peroxidase-conjugated antibodies (GE Healthcare; 1 : 2000) were then added and immunoreactive bands were detected with ECL reagent (GE Healthcare). Quantification of western blots was carried out using ImageJ software and band intensities were normalized to actin levels.

### Immunohistochemistry

Sections (30 *μ*m) of mice brains were analyzed for neuronal inclusions according to the protocol of Davies *et al.*^47^ The sections were labelled with anti-huntingtin antibody by free-floating immunohistochemistry (EM48, Chemicon, Millipore, Watford, UK). Staining was performed by peroxidase labelling using Vectastain avidin:biotinylated enzyme complex (ABC) kit and visualized with DAB reagent (Vector Laboratories, Peterborough, UK).

Inclusions were counted in the piriform cortex and motor cortex in three fields on at least three sections per animal at a magnification of × 100 (Zeiss Axioskop2, field diameter 0.2 mm). Aggregates were photographed and the diameter was measured using Zeiss axiovision software (Zeiss, Cambridge, UK).

Sections (10 *μ*m) of brain were analyzed by hematoxylin (Vector laboratories) and eosin (Sigma) staining. Sections were hydrated, immersed in hematoxylin (45 s), rinsed and placed in 95% ethanol (30 s). Sections were then stained with eosin Y (3 min) followed by graded ethanol submersion (70–100%), before xylene (5 min) and mounting in Depex followed by light microscopy assessment (Zeiss Axioscop 2, MRC5 camera).

### Statistics

Significance levels for comparisons between the groups were determined with *t*-tests, repeated measure, factorial ANOVA, two-way ANOVA or Fisher's exact test, where appropriate, for parametric data and with Mann–Whitney *U*-tests for non-parametric data, for survival curves Log-rank (Mantel–Cox) test was employed. All analyses were carried out using the GraphPad Prism, version 5 (GraphPad Software, La Jolla, CA, USA).

## Abbreviations

CAST: calpastatin
ECL: enhanced chemiluminescence
GFP: green fluorescent protein
HD: Huntington's disease
Htt: huntingtin
LC3: microtubule-associated protein light chain 3
NF449: 4,4′,4′′,4′′′-[carbonylbis[imino-5,1,3-benzenetriylbis(carbonylimino)]]tetrakis-1,3-benzenedisulfonic acid, octasodium salt
PCR: polymerase chain reaction
Q: glutamine
r.p.m: revolutions per minute
RNAi: RNA interference
SDS: sodium dodecyl sulfate
SHIRPA: SmithKline Beecham, Harwell, Imperial College, Royal London Hospital, phenotype assessment
siRNA: small interfering RNA
SOR: spontaneous novel object recognition
Thy1.1: Thymocyte differentiation antigen 1.1
UAS: upstream activating sequence
WT: wild type

## References

[bib1] Imarisio S Carmichael J Korolchuk V Chen CW Saiki S Rose C Huntington's disease: from pathology and genetics to potential therapies Biochem J 2008 412 191 209 18466116 10.1042/BJ20071619

[bib2] Ravikumar B Vacher C Berger Z Davies J Luo S Oroz L Inhibition of mTOR induces autophagy and reduces toxicity of polyglutamine expansions in fly and mouse models of Huntington disease Nat Genet 2004 36 585 595 15146184 10.1038/ng1362

[bib3] Berger Z Ravikumar B Menzies FM Oroz LG Underwood BR Pangalos MN Rapamycin alleviates toxicity of different aggregate-prone proteins Hum Mol Genet 2006 15 433 442 16368705 10.1093/hmg/ddi458

[bib4] Menzies FM Huebener J Renna M Bonin M Riess O Rubinsztein DC Autophagy induction reduces mutant ataxin-3 levels and toxicity in a mouse model of spinocerebellar ataxia type 3 Brain 2010 133 (Pt 1 93 104 20007218 10.1093/brain/awp292 PMC2801325

[bib5] Menzies FM Moreau K Rubinsztein DC Protein misfolding disorders and macroautophagy Curr Opin Cell Biol 2011 23 190 197 21087849 10.1016/j.ceb.2010.10.010 PMC3080604

[bib6] Ravikumar B Duden R Rubinsztein D Aggregate-prone proteins with polyglutamine and polyalanine expansions are degraded by autophagy Hum Mol Genet 2002 11 1107 1117 11978769 10.1093/hmg/11.9.1107

[bib7] Ono Y Sorimachi H Calpains: an elaborate proteolytic system Biochim Biophys Acta 2012 1824 224 236 21864727 10.1016/j.bbapap.2011.08.005

[bib8] Williams A Sarkar S Cuddon P Ttofi E Saiki S Siddiqi F Novel targets for Huntington's disease in an mTOR-independent autophagy pathway Nat Chem Biol 2008 4 295 305 18391949 10.1038/nchembio.79 PMC2635566

[bib9] Yousefi S Perozzo R Schmid I Ziemiecki A Schaffner T Scapozza L Calpain-mediated cleavage of Atg5 switches autophagy to apoptosis Nat Cell Biol 2006 8 1124 1132 16998475 10.1038/ncb1482

[bib10] Xia HG Zhang L Chen G Zhang T Liu J Jin M Control of basal autophagy by calpain1 mediated cleavage of ATG5 Autophagy 2010 6 61 66 19901552 10.4161/auto.6.1.10326 PMC2883879

[bib11] Friedrich P Tompa P Farkas A The calpain-system of *Drosophila melanogaster*: coming of age Bio Essays 2004 26 1088 1096 10.1002/bies.20106 15382138

[bib12] Martindale D Hackam A Wieczorek A Ellerby L Wellington C McCutcheon K Length of huntingtin and its polyglutamine tract influences localization and frequency of intracellular aggregates Nat Genet 1998 18 150 154 9462744 10.1038/ng0298-150

[bib13] Wellington CL Singaraja R Ellerby L Savill J Roy S Leavitt B Inhibiting caspase cleavage of huntingtin reduces toxicity and aggregate formation in neuronal and nonneuronal cells J Biol Chem 2000 275 19831 19838 10770929 10.1074/jbc.M001475200

[bib14] Gafni J Ellerby LM Calpain activation in Huntington's disease J Neurosci 2002 22 4842 4849 12077181 10.1523/JNEUROSCI.22-12-04842.2002 PMC6757710

[bib15] Gafni J Hermel E Young JE Wellington CL Hayden MR Ellerby LM Inhibition of calpain cleavage of huntingtin reduces toxicity: accumulation of calpain/caspase fragments in the nucleus J Biol Chem 2004 279 20211 20220 14981075 10.1074/jbc.M401267200

[bib16] Landles C Sathasivam K Weiss A Woodman B Moffitt H Finkbeiner S Proteolysis of mutant huntingtin produces an exon 1 fragment that accumulates as an aggregated protein in neuronal nuclei in Huntington disease J Biol Chem 2010 285 8808 8823 20086007 10.1074/jbc.M109.075028 PMC2838303

[bib17] Zhang S Binari R Zhou R Perrimon N A genomewide RNA interference screen for modifiers of aggregates formation by mutant Huntingtin in *Drosophila* Genetics 2010 184 1165 1179 20100940 10.1534/genetics.109.112516 PMC2865916

[bib18] Simonsen A Cumming RC Brech A Isakson P Schubert DR Finley KD Promoting basal levels of autophagy in the nervous system enhances longevity and oxidant resistance in adult *Drosophila* Autophagy 2008 4 176 184 18059160 10.4161/auto.5269

[bib19] Franceschini N Kirschfeld K [Pseudopupil phenomena in the compound eye of drosophila] Kybernetik 1971 9 159 182 5134358 10.1007/BF02215177

[bib20] Jackson GR Salecker I Dong X Yao X Arnheim N Faber PW Polyglutamine-expanded human huntingtin transgenes induce degeneration of *Drosophila* photoreceptor neurons Neuron 1998 21 633 642 9768849 10.1016/s0896-6273(00)80573-5

[bib21] Marsh JL Walker H Theisen H Zhu YZ Fielder T Purcell J Expanded polyglutamine peptides alone are intrinsically cytotoxic and cause neurodegeneration in *Drosophila* Hum Mol Genet 2000 9 13 25 10587574 10.1093/hmg/9.1.13

[bib22] Reinecke JB DeVos SL McGrath JP Shepard AM Goncharoff DK Tait DN Implicating calpain in tau-mediated toxicity *in vivo* PLoS One 2011 6 e23865 21858230 10.1371/journal.pone.0023865 PMC3157467

[bib23] Wittmann CW Wszolek MF Shulman JM Salvaterra PM Lewis J Hutton M Tauopathy in *Drosophila*: neurodegeneration without neurofibrillary tangles Science 2001 293 711 714 11408621 10.1126/science.1062382

[bib24] Rao MV Mohan PS Peterhoff CM Yang DS Schmidt SD Stavrides PH Marked calpastatin (CAST) depletion in Alzheimer's disease accelerates cytoskeleton disruption and neurodegeneration: neuroprotection by CAST overexpression J Neurosci 2008 28 12241 12254 19020018 10.1523/JNEUROSCI.4119-08.2008 PMC2819018

[bib25] Mangiarini L Sathasivam K Seller M Cozens B Harper A Hetherington C Exon 1 of the HD gene with an expanded CAG repeat is sufficient to cause a progressive neurological phenotype in transgenic mice Cell 1996 87 493 506 8898202 10.1016/s0092-8674(00)81369-0

[bib26] Rogers DC Fisher EM Brown SD Peters J Hunter AJ Martin JE Behavioral and functional analysis of mouse phenotype: SHIRPA, a proposed protocol for comprehensive phenotype assessment Mamm Genome 1997 8 711 713 9321461 10.1007/s003359900551

[bib27] Kastenmayer RJ Fain MA Perdue KA A retrospective study of idiopathic ulcerative dermatitis in mice with a C57BL/6 background J Am Assoc Lab Ani Sci 2006 45 8 12 17089984

[bib28] Narain Y Wyttenbach A Rankin J Furlong RA Rubinsztein DC A molecular investigation of true dominance in Huntington's disease J Med Genet 1999 36 739 746 10528852 10.1136/jmg.36.10.739 PMC1734229

[bib29] Saito K Elce JS Hamos JE Nixon RA Widespread activation of calcium-activated neutral proteinase (calpain) in the brain in Alzheimer disease: a potential molecular basis for neuronal degeneration Proc Natl Acad Sci USA 1993 90 2628 2632 8464868 10.1073/pnas.90.7.2628 PMC46148

[bib30] Sepers MD Raymond LA Mechanisms of synaptic dysfunction and excitotoxicity in Huntington's disease Drug Discov Today 2014 19 990 996 24603212 10.1016/j.drudis.2014.02.006

[bib31] Siman R Card JP Davis LG Proteolytic processing of beta-amyloid precursor by calpain I J Neurosci 1990 10 2400 2411 2115911 10.1523/JNEUROSCI.10-07-02400.1990 PMC6570374

[bib32] Mercken M Grynspan F Nixon RA Differential sensitivity to proteolysis by brain calpain of adult human tau, fetal human tau and PHF-tau FEBS Lett 1995 368 10 14 7615058 10.1016/0014-5793(95)00590-6

[bib33] Yang LS Ksiezak-Reding H Calpain-induced proteolysis of normal human tau and tau associated with paired helical filaments Eur J Biochem 1995 233 9 17 7588778 10.1111/j.1432-1033.1995.009_1.x

[bib34] Rao MV McBayer MK Campbell J Kumar A Hashim A Sershen H Specific calpain inhibition by calpastatin prevents tauopathy and neurodegeneration and restores normal lifespan in Tau P301L Mice J Neurosci 2014 34 9222 9234 25009256 10.1523/JNEUROSCI.1132-14.2014 PMC4087203

[bib35] Dufty BM Warner LR Hou ST Jiang SX Gomez-Isla T Leenhouts KM Calpain-cleavage of alpha-synuclein: connecting proteolytic processing to disease-linked aggregation Am J Pathol 2007 170 1725 1738 17456777 10.2353/ajpath.2007.061232 PMC1854966

[bib36] Diepenbroek M Casadei N Esmer H Saido TC Takano J Kahle PJ Overexpression of the calpain-specific inhibitor calpastatin reduces human alpha-Synuclein processing, aggregation and synaptic impairment in [A30P]alphaSyn transgenic mice Hum Mol Genet 2014 23 3975 3989 24619358 10.1093/hmg/ddu112 PMC4110482

[bib37] Webb JL Ravikumar B Atkins J Skepper JN Rubinsztein DC Alpha-Synuclein is degraded by both autophagy and the proteasome J Biol Chem 2003 278 25009 25013 12719433 10.1074/jbc.M300227200

[bib38] Dietzl G Chen D Schnorrer F Su KC Barinova Y Fellner M A genome-wide transgenic RNAi library for conditional gene inactivation in *Drosophila* Nature 2007 448 151 156 17625558 10.1038/nature05954

[bib39] Freeman M Reiterative use of the EGF receptor triggers differentiation of all cell types in the *Drosophila* eye Cell 1996 87 651 660 8929534 10.1016/s0092-8674(00)81385-9

[bib40] Perrin L Bloyer S Ferraz C Agrawal N Sinha P Dura JM The leucine zipper motif of the *Drosophila* AF10 homologue can inhibit PRE-mediated repression: implications for leukemogenic activity of human MLL-AF10 fusions Mole Cel Biol 2003 23 119 130 10.1128/MCB.23.1.119-130.2003 PMC140655 12482966

[bib41] Dimitroff B Howe K Watson A Campion B Lee HG Zhao N Diet and energy-sensing inputs affect TorC1-mediated axon misrouting but not TorC2-directed synapse growth in a *Drosophila* model of tuberous sclerosis PLoS One 2012 7 e30722 22319582 10.1371/journal.pone.0030722 PMC3272037

[bib42] Lin DM Goodman CS Ectopic and increased expression of Fasciclin II alters motoneuron growth cone guidance Neuron 1994 13 507 523 7917288 10.1016/0896-6273(94)90022-1

[bib43] Simonsen A Cumming R Brech A Isakson P Schubert D Finley K Promoting basal levels of autophagy in the nervous system enhances longevity and oxidant resistance in adult *Drosophila* Autophagy 2008 4 176 184 18059160 10.4161/auto.5269

[bib44] Green EW Fedele G Giorgini F Kyriacou CP A *Drosophila* RNAi collection is subject to dominant phenotypic effects Nat Methods 2014 11 222 223 24577271 10.1038/nmeth.2856

[bib45] Schilling G Becher MW Sharp AH Jinnah HA Duan K Kotzuk JA Intranuclear inclusions and neuritic aggregates in transgenic mice expressing a mutant N-terminal fragment of huntingtin Hum Mole Genet 1999 8 397 407 10.1093/hmg/8.3.397 9949199

[bib46] Romberg C McTighe SM Heath CJ Whitcomb DJ Cho K Bussey TJ False recognition in a mouse model of Alzheimer's disease: rescue with sensory restriction and memantine Brain 2012 135 (Pt 7 2103 2114 22466291 10.1093/brain/aws074 PMC3381719

[bib47] Davies SW Sathasivam K Hobbs C Doherty P Mangiarini L Scherzinger E Detection of polyglutamine aggregation in mouse models Methods Enzymol 1999 309 687 701 10507055 10.1016/s0076-6879(99)09045-x

