# Supplementary figures and images for: Calpain inhibition mediates autophagy-dependent protection against polyglutamine toxicity
Source: Cell Death Differ. 2014 Sep 26;22(3):433–44. doi: 10.1038/cdd.2014.151 (PMC4326573; doi:10.1038/cdd.2014.151)

## Slide 1
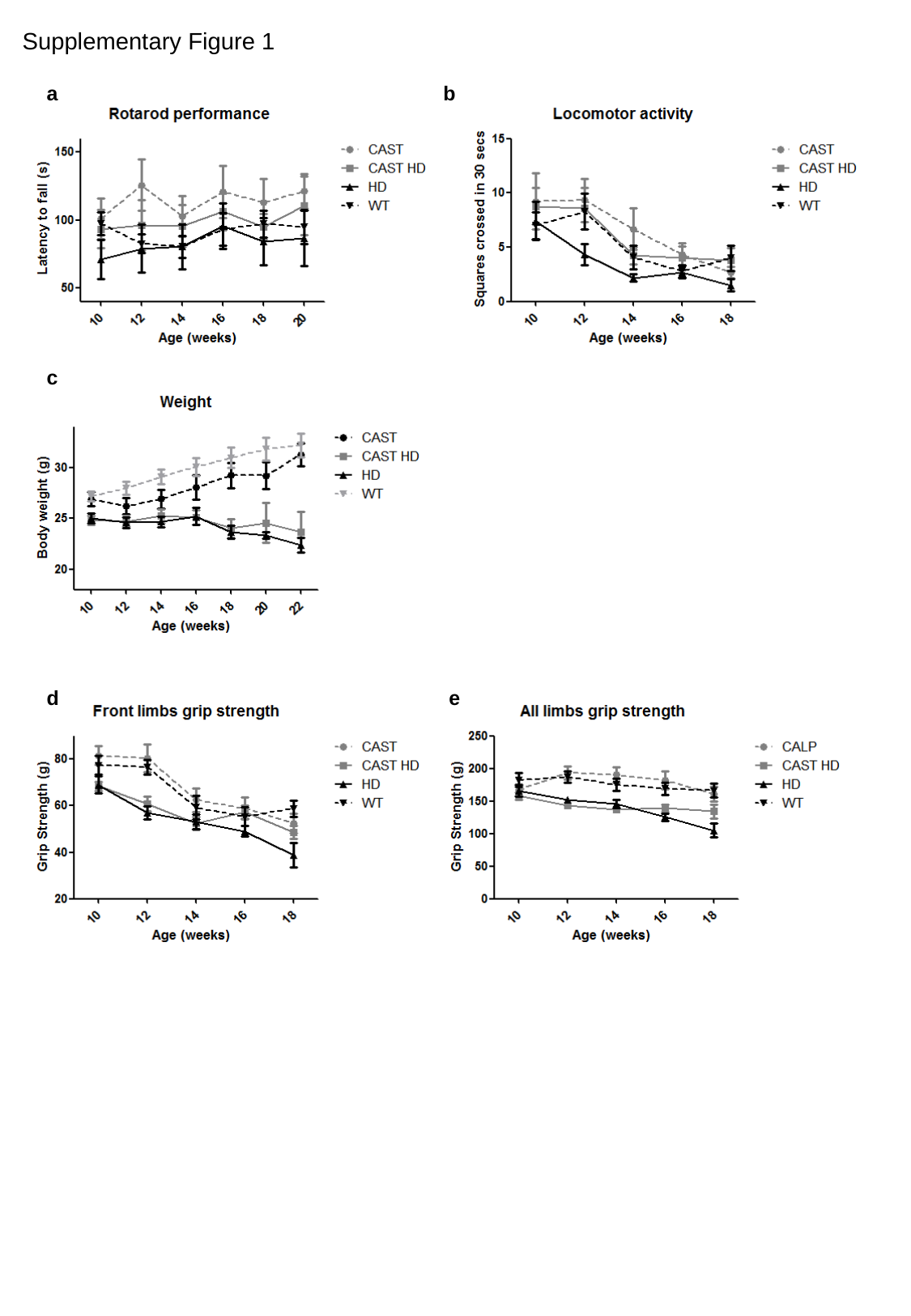

Supplementary Figure 1
a
b
c
d
e

## Slide 2
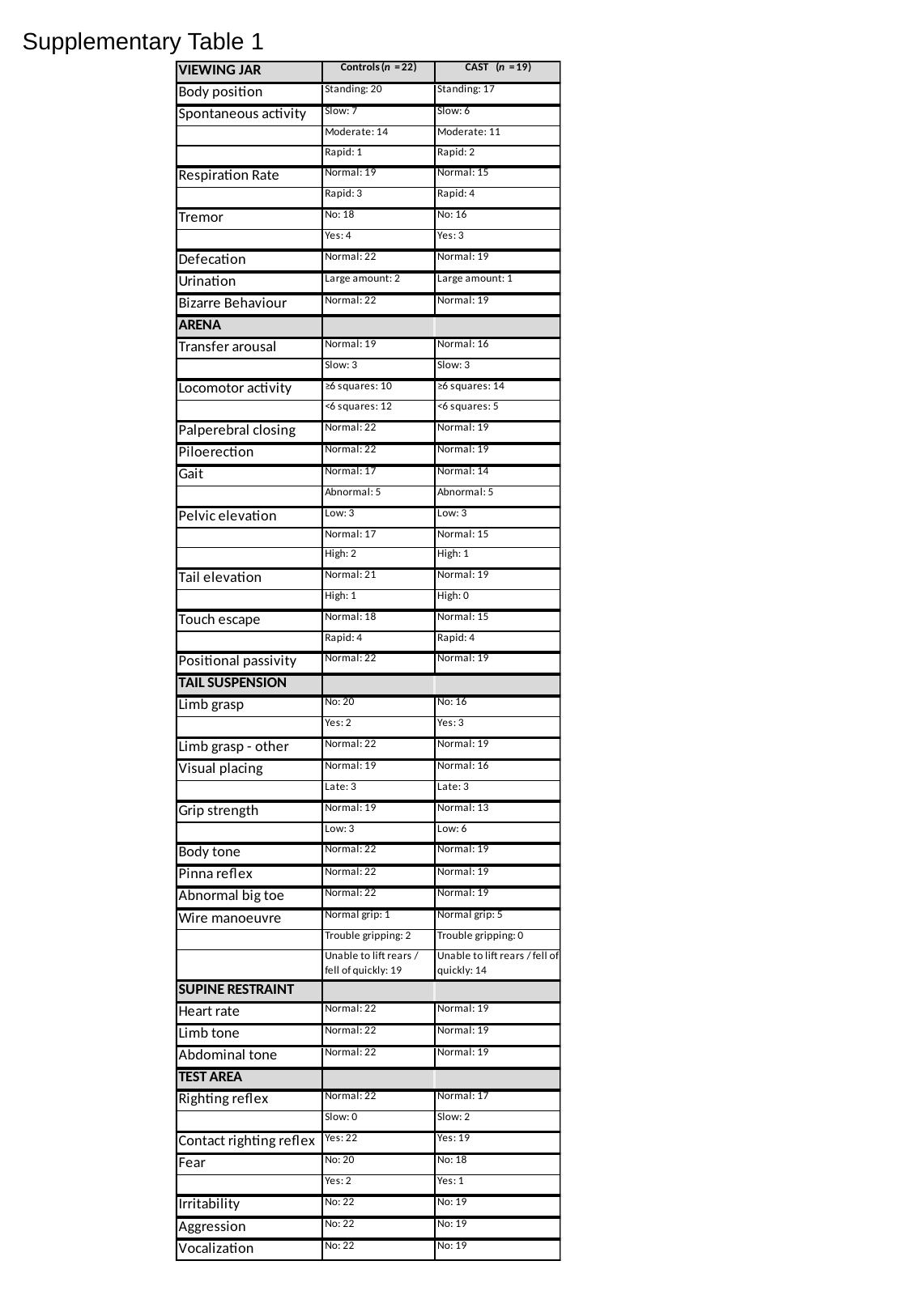

Supplementary Table 1

Supplement: Supplementary Figure [file cdd2014151x1.ppt]
