# Supplementary material for: Calpain inhibition mediates autophagy-dependent protection against polyglutamine toxicity
Source: Cell Death Differ. 2014 Sep 26;22(3):433–44. doi: 10.1038/cdd.2014.151 (PMC4326573; doi:10.1038/cdd.2014.151)
Supplement: Supplementary Figure Legend [file cdd2014151x2.doc]

**Supplementary Figure 1:** Complete datasets for all genotypes used in calpastatin overexpression study in N171-82Q mice. (**a**) Rotarod performance shown as mean best performance from three trial runs for each mouse. Note that wild-type mice do not perform better than HD mice from 14 weeks old, this is likely due to the increased weight of the wild-type animals (**b**) Locomotor activity measured by open field test (**c**) Body weight of mice from the four genotypes assessed (**d**) Forelimb grip strength (**e**) Grip strength of all limbs together. In all graphs, black lines represent N171-82Q, single transgenic HD mice (HD), dotted black lines represent non-transgenic littermate control mice (WT), grey lines represent double transgenic calpastatin and N171-82Q Htt overexpressing mice (CAST HD) and dotted grey lines represent single transgenic calpastatin overexpressing mice (CAST), error bars represent SEM.

**Supplementary Table 1:** SHIRPA scores for aged CAST and WT mice. The results of SHIRPA phenotyping for aged mice is summarized in the table. For each of the phenotypes assessed the number of mice within each category is shown. Only categories in which mice were observed are listed.
